# Quantifying Anuran Microhabitat Use to Infer the Potential for Parasite Transmission between Invasive Cane Toads and Two Species of Australian Native Frogs

**DOI:** 10.1371/journal.pone.0106996

**Published:** 2014-09-04

**Authors:** Lígia Pizzatto, Camila Both, Richard Shine

**Affiliations:** 1 School of Biological Sciences, University of Sydney, Sydney, Australia; 2 Programa de Pós Graduação em Zoologia, Pontifícia Universidade Católica do Rio Grande do Sul, Porto Alegre, Rio Grande do Sul, Brazil; Leibniz-Institute of Freshwater Ecology and Inland Fisheries, Germany

## Abstract

Parasites that are carried by invasive species can infect native taxa, with devastating consequences. In Australia, invading cane toads (*Rhinella marina*) carry lungworm parasites (*Rhabdias pseudosphaerocephala*) that (based on previous laboratory studies) can infect native treefrogs (*Litoria caerulea* and *L. splendida*). To assess the potential of parasite transmission from the invader to the native species (and from one infected native frog to another), we used surveys and radiotelemetry to quantify anuran microhabitat use, and proximity to other anurans, in two sites in tropical Australia. Unsurprisingly, treefrogs spent much of their time off the ground (especially by day, and in undisturbed forests) but terrestrial activity was common at night (especially in anthropogenically modified habitats). Microhabitat overlap between cane toads and frogs was generally low, except at night in disturbed areas, whereas overlap between the two frog species was high. The situations of highest overlap, and hence with the greatest danger of parasite transmission, involve aggregations of frogs within crevices by day, and use of open ground by all three anuran species at night. Overall, microhabitat divergence between toads and frogs should reduce, but not eliminate, the transmission of lungworms from invasive toads to vulnerable native frogs.

## Introduction

Invasive species can affect native biota by several pathways, such as predation [1], changing habitat structure [2], or the provision of a novel food source [3]. An important but poorly-understood mechanism involves the transmission of parasites and diseases from an invader to native taxa [4]. For example, chytrid fungus has caused amphibian declines worldwide [5], and parasites have caused widespread mortality of European fishes [6], [7]. Similar transfers of pathogens continue to be reported [8]. Whether or not a native species is infected and impacted by an invader’s parasite depends upon a suite of physiological, behavioral, and ecological parameters that influence host-parasite compatibility [9], [10]. On a physiological level, the immune system of the novel host may destroy any propagules from the newly-encountered parasite. Similarly, the native taxa may resist parasite uptake (e.g., pigeon lice are successfully removed by preening, and do not colonize novel hosts that are smaller than original hosts [11]). On an ecological level, differences in microhabitat use by invaders versus native species may restrict parasite transmission, especially if the infective stages of the parasite are short-lived, or require specific abiotic conditions.

The possibility of parasite transfer during the spread of cane toads (*Rhinella* (*Bufo*) *marina*) through tropical Australia has raised significant concern. The toads have brought nematode lungworms (*Rhabdias pseudosphaerocephala*) with them from their native range in the Americas [12]. Adult hermaphroditic nematodes live in the host’s lungs, and produce eggs that hatch into L1 larvae. After being defecated, L1 larvae molt into L2 and then infective larvae (L3) or free-living dioecious adults that also produce L3 larvae [13]. L3 live in the soil, and directly penetrate new anuran hosts, via the skin or over the eyeball [13]–[15], then migrate to the lungs. These lungworms can infect native frogs [15], but typically do not survive longterm inside Australian anurans [16]. However, laboratory studies show that two species of large treefrogs can sustain high infection levels of adult worms in the lungs [17]. One of these species (the Green Treefrog, *Litoria caerulea*) shows no overt ill effects from the infections whereas the other (Magnificent Treefrog, *L. splendida*) exhibits high levels of mortality [17]. Thus, an absence of records of the toad lungworm in Australian native frogs from surveys [18] might reflect either a lack of transmission, or high mortality of infected frogs. The cane toad invasion has already spread through large areas containing *L. caerulea*, and has now reached areas containing *L. splendida*.

Will the cane toad transfer its lethal lungworms to the vulnerable native taxon, either directly (toad to Magnificent Treefrog) or indirectly (toad to Green Treefrog to Magnificent Treefrog)? Those outcomes (as well as other mechanisms of invader impact, such as predation and competition) will depend upon microhabitat overlap, and proximity of individuals of the three species. To quantify these parameters, we conducted studies at two sites: one in the Northern Territory (where toads and Green Treefrogs co-occur), and one in Western Australia (where all three species are sympatric). Using standardized surveys as well as radio-tracking, we asked:

1. What microhabitat types are used by these anuran taxa, by day and by night, in disturbed versus natural environments, in both of our study sites?
2. To what degree does arboreal behavior by treefrogs reduce microhabitat overlap between toads and frogs?
3. How often are toads and frogs found in close proximity? and
4. Based on proximity and microhabitat overlap, are native species likely to overlap in habitat use with infected toads?

## Methods

### Ethics Statement

This study was conducted under ethical approval from the University of Sydney Animal Ethics Committee (L04/5-2010/2/5334), and approvals from the Northern Territory Parks and Wildlife (39857) and Western Australian Department of Environment and Conservation (SF007610). The owners or their representatives provided access to private lands.

### Study Species

**Figure 1:**
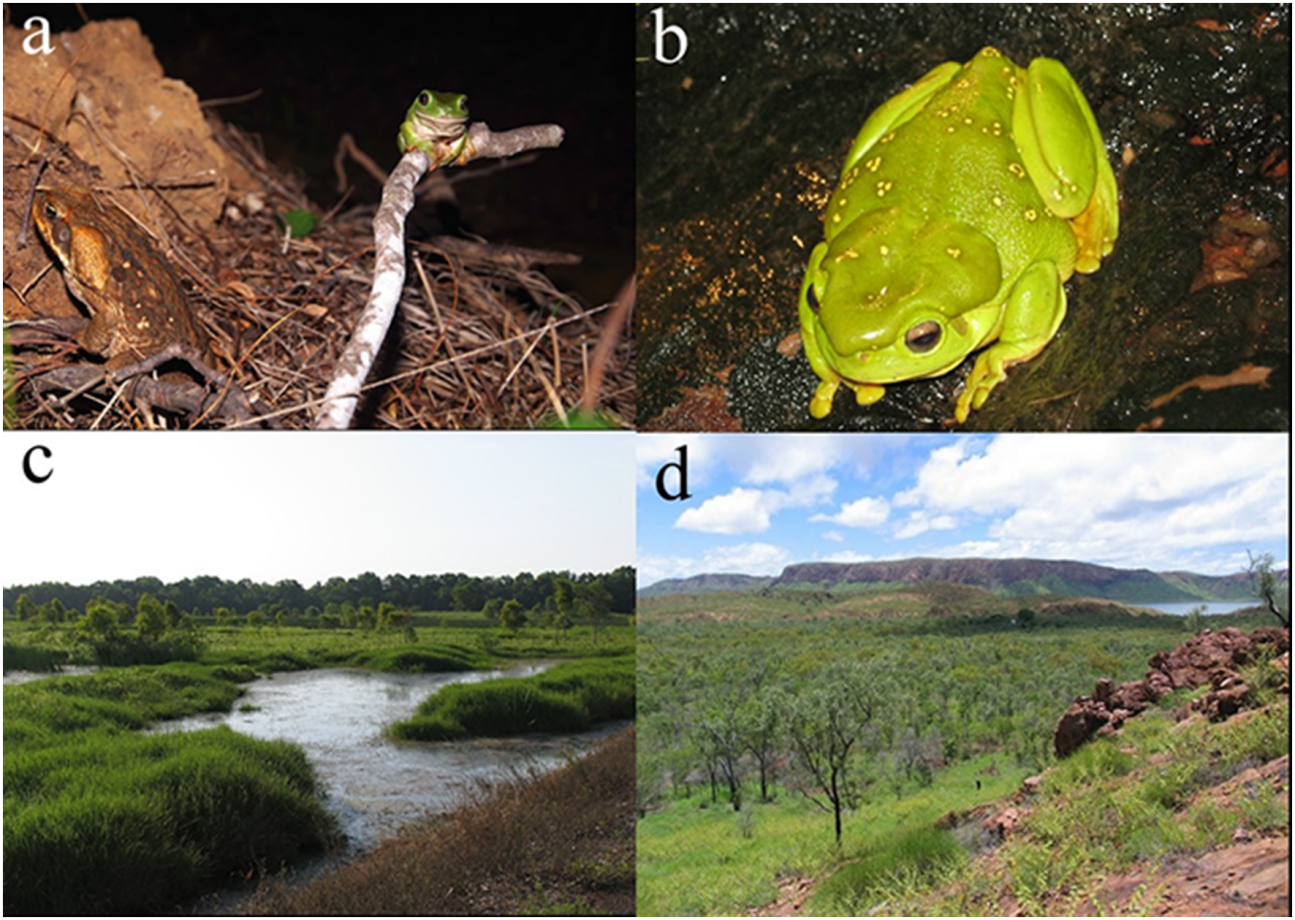
Study species and areas. (A) A cane toad *Rhinella marina* on leaf litter beside a Green Treefrog *Litoria caerulea* on a fallen branch in the Northern Territory. (B) A Magnificent Treefrog (*L. splendida*) on a rock outcrop in the Kimberley, Western Australia. (C) Habitat in the Adelaide River floodplain and adjacent monsoon forest (in the background), in Fogg Dam Conservation Reserve, Northern Territory. (D) Woodlands and limestone escarpments surrounding Lake Argyle in the Kimberley region, Western Australia.

Green Treefrogs (*Litoria caerulea*) and Magnificent Treefrogs (*L. splendida*) are morphologically similar, and closely related [19]. They are bright green, heavy-bodied, and among the largest Australian anurans (to 110 mm: Figure 1a,b). *Litoria caerulea* is broadly distributed in the north and east of Australia whereas *L. splendida* is restricted to the Kimberley region (north-eastern Western Australia) and adjacent Keep River National Park in the Northern Territory [20]. The cane toad *Rhinella marina* is an even larger terrestrial anuran (to 240 mm: Figure 1a) native to South and Central America, and widely translocated around the world in attempts to control insect pests [21]. Cane toads were introduced to Queensland (eastern Australia) in 1935; they are now abundant throughout most of tropical Australia [22], including arid areas [23]. Adults of all three anuran species are nocturnally active, with cane toads moving about and feeding virtually year-round, except in prolonged dry periods [24], whereas most native anurans are inactive for long periods during the dry-season [25]. Our surveys and radio-tracking primarily were carried out during the wet-season, when all of our study taxa are active and feeding (and hence, when infected animals are likely to void parasite larvae in their feces).

### Study Sites and Sampling

#### Northern Territory (NT)

Cane toads (*R. marina*) and Green Treefrogs (*L. caerulea*) were sampled in the monsoon forest in Fogg Dam Conservation Reserve (Figure 1c) and adjacent rural settlements in the Adelaide River floodplain area, 60 km southeast of Darwin (12°34′10″S, 131°18′43″E). The topography is generally flat. In the monsoon forest we delimited two transects of 600 m along a pre-established track, and sampled all anurans within 3 m on each side of the track (i.e., in a total area of 3600 m^2^). In three anthropogenically-disturbed sites (12°34′43″E, 131°18′52″E; 12°39′04″S, 131°19′06″E; 12°38′49″S, 131°19′00″E), we surveyed around buildings and cattle yards (areas of 36.7, 51.5 and 28.2 m^2^). All five sites were surveyed one to three times (to obtain data on sufficient numbers of anurans) during a season. Repeat surveys at a site were conducted 1–6 weeks apart. We obtained additional records during other work, while walking and driving near the edges of the monsoon forests and in anthropogenically disturbed areas. Surveys were conducted at night (1900–2340 h), with some occasional encounters during the day (1000–1250 h). Data were collected September to December 2010, April 2011, and December to January 2012.

#### Western Australia (WA)

Our second study site was in the eastern Kimberley, on the northern side of Lake Argyle (Figure 1d; 2 years post-toad-invasion), and around the town of Kununurra (as toads were first invading). This area experiences a similar wet-dry climate (monsoon-dominated) as the NT site, but the terrain is rocky, with sandstone and limestone gorges among open woodland. We sampled cane toads, Green Treefrogs and Magnificent Treefrogs, during nocturnal surveys (1900–2240 h) from December 2010 to March 2011. We searched several times through each of three natural areas: a rocky outcrop covering 9784 m^2^ (16°06′87″S, 128°44′47″E), a transect in the open savannah covering 1182 m^2^ (16°06′29″S, 128°44′50″E), and a cave and adjacent savannah (360 m transect, between the towns of Kununurra and Wyndham 15°46′09″S, 128°39′16″E). The anthropogenically-disturbed areas were around a building in the savannah (1756 m^2^; 16°06′34″S, 128°44′59″E), and a caravan park (21782 m^2^; 16°06′47″S, 128°44′26″E). We also recorded occasional encounters with anurans while walking around those areas. For safety reasons, the remote sites were only surveyed by day (0750–1400 h).

### Radio-tracking

In anurans, the host behaviors that affect parasite transmission (such as microhabitat use and aggregation) often differ between day and night. Thus, we need data for both time periods. Locating inactive anurans by day (inside their shelter sites) is difficult, but can be achieved by radiotelemetry. Radio-tracking also can provide more detailed and reliable data on microhabitat use than can surveys, because radio-tagged animals can be located even in habitats where visual observation is impossible. Accordingly, we radio-tracked adults of the three species of anurans. Radio-transmitters (Holohil, BD-2 and PD-2, 1.56 and 3.13 g, customized for belt attachment) were sewn onto a soft fabric belt. Anurans were captured by hand, weighed, and their sex was recorded. A belt was tied around the animal’s waist, with the antenna directed backwards. The radio-transmitter and belt pack weighed less than 5% of anuran body mass, and the animals were released at the site of capture within a few minutes. We disinfected our hands with ethanol gel between successive captures. The anurans were located twice daily (day and night), except during extreme weather events, and the duration of tracking per individual was determined by logistical factors. At each location, we recorded the same habitat variables as we did during surveys.

### Microhabitat Variables

For each individual seen, we recorded the GPS location, date, time of the encounter, air temperature, raining or not, air humidity, type of environment (anthropogenically disturbed vs. natural), type and height above ground of substrate used by the anuran, and distance to the nearest water source. Microhabitat types were scored into seven major categories: ‘arboreal’ (branches, fences, roofs, tables, walls), ‘crevices’ (in rocks or artificial, such as inside pipes and poles), on ‘grass or leaf litter’, on ‘open ground’ (pavements, rocky or bare ground), ‘under vegetation’ (including under grass and leaf litter), or ‘in water’. We also recorded whether or not frogs were exhibiting breeding activity (calling or amplexus), nearest neighbor species (of the three study species only), and distance to the nearest neighbor (if >50 m, we scored the radio-tracked animal as ‘solitary’). For frogs in amplexus, the partner was not included in the calculation of nearest neighbor. Distance from water was categorized as: ‘in water’ (0–2 m from water), ‘very near’ (2.01–5 m), ‘near’ (5.01–9.99 m), ‘medium’ (10–20 m), and ‘far’ (>20 m). Data on rainfall were obtained from the Australian Bureau of Meteorology (www.bom.gov.au) for the Fogg Dam Area (Middle Point weather station) and Kununurra (airport weather station), and measured directly at the Lake Argyle area.

### Data Analyses

#### Microhabitat use

We compiled a matrix containing microhabitat data from all individuals found in surveys and occasional encounters. For each radio-tracked individual, we only included one nocturnal and one diurnal observation (randomly selected). We tested if the three species differed in microhabitat use through permutational multivariate analysis of variance [26], with 10000 permutations, using the software Multiv [27]. We used the Gower index [28] as our measure of similarity. Substrate type and height above ground were used as microhabitat descriptors; species, environment type (natural/anthropogenic) and time (day/night) were the fixed factors. Permutations were blocked within each study area, and differences between species pairs were computed by pairwise contrasts [26].

We estimated microhabitat overlap by calculating Pianka’s index of niche overlap [29] for all species, and each species pair, comparing the proportion of use of microhabitat types. Microhabitat type was defined based on substrate type and height above ground (<30 cm, 31–70 cm, >70 cm). Indices were calculated for each combination of study area, type of environment (disturbed vs. natural) and time of encounter (day/night). Pianka’s index can range from 0 (no niche overlap) to 1 (complete overlap). As before, we pooled data for radio-tracked individuals plus occasional encounters and surveys, using only one record for each radio-tracked individual to avoid non-independence in the data. Indices were calculated to quantify the degree of overlap, to facilitate comparison between environments and time periods, and to allow comparison with other studies.

To test whether or not species overlap was more (or less) in microhabitat use than expected by chance, we compared the observed overlap indices with those obtained from 1000 randomized matrices in a null model. Null matrices were built using a relaxed niche breadth algorithm (which relaxes the degree of specialization of each species) and allowing reshuffle of the zeros (which allows a species to use a substrate type that was never observed being used in the wild) [30]. Cases where a species could not possibly use a specific substrate (e.g., toads on arboreal branches) were scored as ‘hard zeros’ for that species. Hard zeros are not reshuffled in simulated matrices [30]. Analyses were carried out using the function ‘niche overlap’ in the software EcoSim 7.71 (http://www.garyentsminger.com/ecosim/index.htm).

#### Nearest neighbor analyses

Using data from our nocturnal surveys and occasional encounters, we conducted nominal logistic regression to analyze the effects of region (NT or WA sites), climatic variables, distance to water, time of encounter (nocturnal data only), type of environment (natural vs. disturbed), and interactions among those variables, on the identity of the nearest neighbor pair of species. Non-significant interaction terms were deleted from the final model. We used generalized linear modeling (GLM) with a negative binomial distribution to model the distance to the nearest neighbor as a function of region, climatic variables, species pair of nearest neighbors, distance to water, type of environment, time of encounter, and interactions among those variables. Climatic data in each region were combined in a Principal Component Analyses (PCA) due to high autocorrelation among the variables. PC1 explained 53.4% of the variation and was used in the GLMs when all three climatic variables had a significant effect on the response variable. Too few data were gathered on nearest neighbors during the day (n = 45) to warrant analysis, so we only used data from nocturnal observations.

To calculate nearest-neighbor relationships, we analyzed data from radio-tracked animals separately from the survey and ‘occasional encounter’ animals. Repeated observation on the radio-tagged anurans otherwise would compromise non-independence; and randomly selecting only one data point per animal (as we did for microhabitat analyses) would result in too much loss of information, because radio-tracked individuals varied more in nearest neighbor identity and distance than in microhabitat use. For analyses on the identity of the nearest neighbor, we calculated the proportion of times each species was recorded as the nearest neighbor of each tracked individual. We compared the proportions of neighbor-species pairs using the Kruskall-Wallis test, on pooled data from natural and disturbed environments. To quantify ‘distance to nearest neighbor’ of radio-tracked individuals, we used the average distances per individual to minimize dependence, and time of encounter was categorized as ‘day or night’ rather than a continuous variable. Climatic variables were excluded from this analysis, because they did not have significant effects nor improve the fit of the model. We used R (http://www.r-project.org/) to calculate GLMs, and JMP 9.0 (SAS Institute, Cary, NC) to conduct logistic regressions, Kruskal-Wallis tests and PCA.

## Results

At the NT study site we obtained 318 nocturnal records (135 for toads, 183 for Green Treefrogs) and sixteen diurnal records (all of Green Treefrogs). At the WA site we obtained 241 nocturnal records (93 toads, 63 Green Treefrogs, and 85 Magnificent Treefrogs), plus 67 diurnal records (19 Green Treefrogs, 48 Magnificent Treefrogs).

At the NT site we radio-tracked 12 toads and 13 Green Treefrogs, and at the WA site we tracked 23 toads, 9 Green Treefrogs, and 16 Magnificent Treefrogs. The duration of tracking varied from 3–10 days, depending on logistics (e.g., WA toads had brief tracking periods because they moved extensively, often to inaccessible sites).

### Microhabitat Use and Overlap

**Figure 2:**
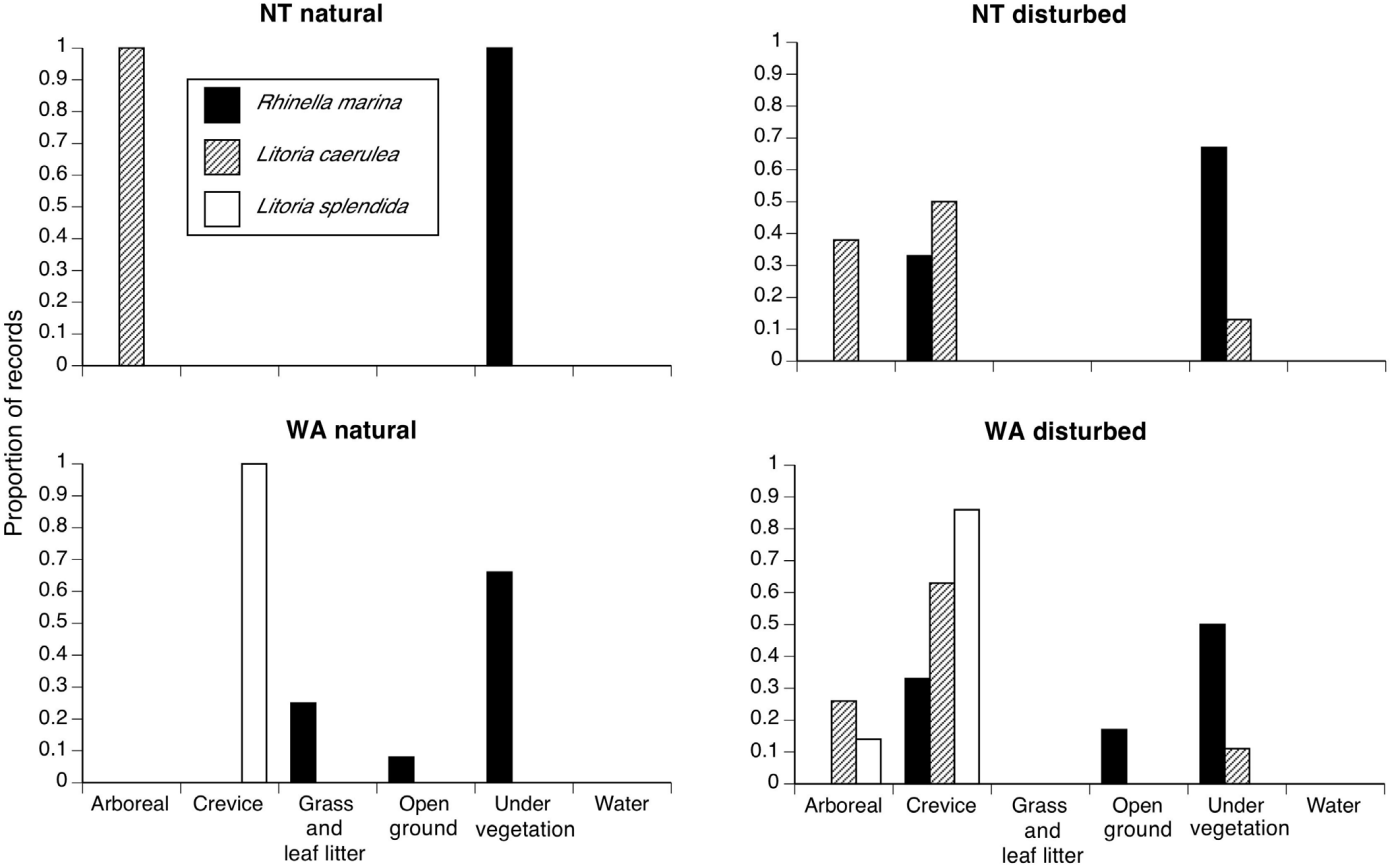
Diurnal substrates used by the three anurans in NT and WA. Cane toads (*Rhinella marina* = black bars), Green Treefrogs (*Litoria caerulea* = light grey bars), and Magnificent Treefrogs (*L. splendida* = dark grey bars) were sampled in natural and anthropogenically disturbed environments. Data for radio-tracked animals were included as only one record per period per individual.

**Figure 3:**
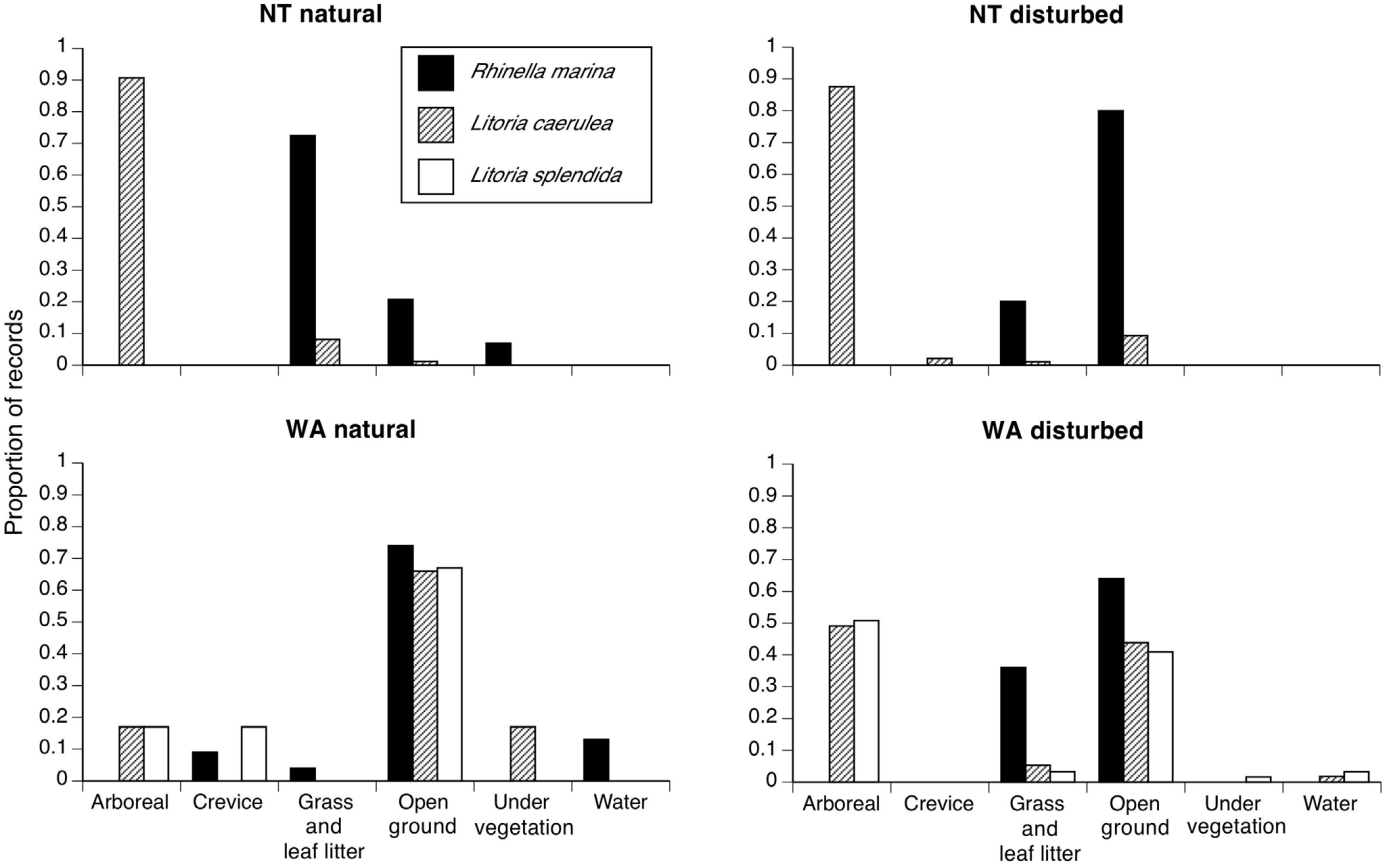
Nocturnal substrates used by the three anurans in NT and WA. Cane toads (*Rhinella marina* = black bars), Green Treefrogs (*Litoria caerulea* = light grey bars), and Magnificent Treefrogs (*L. splendida* = dark grey bars) were sampled in natural and anthropogenically disturbed environments. For radio-tracked animals, only a single record per tracking period was included for each individual.

**Figure 4:**
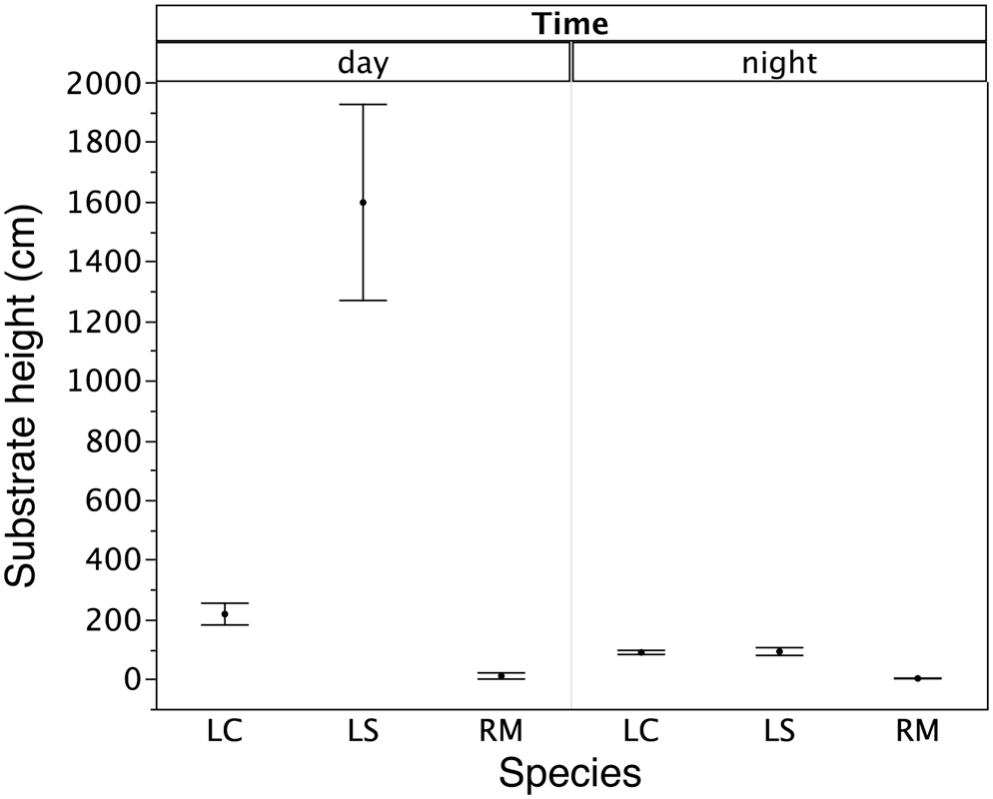
Height above ground of substrate used by the three anurans in NT and WA. Cane toads (*R. marina*) = RM, green tree frogs (*L. caerulea*) = LC, and magnificent tree frogs (*L. splendida*) = LS. Circles represent averages and bars are standard errors. Data for both study areas and environments are pooled together.

At both of our study sites, cane toads were generally found sheltering under vegetation by day (Figure 2, Table S1 in File S1), and on open ground (often, paved roads or bare soil) or on grass and leaf litter by night (Figure 3, Tables S1 and S2 in File S1). In anthropogenically disturbed habitats, some toads selected crevices (under rocks, buildings, logs, etc.) as diurnal shelters (Figure 2, Tables S1 and S2 in File S1). Treefrogs were generally located inside crevices or in arboreal perches by day, although *L. caerulea* at disturbed sites sometimes spent the day beneath vegetation on the ground (Figure 2). At night we found treefrogs on the ground (often, moving across open areas such as paved roads, or on pavements around buildings) as well as in arboreal perches (Figure 3). Both frog species consistently used substrates that were higher off the ground than those used by toads in both regions (and in both disturbed and natural areas), especially during the day (Figure 4, Tables S1 and S2 in File S1).

**Figure 5:**
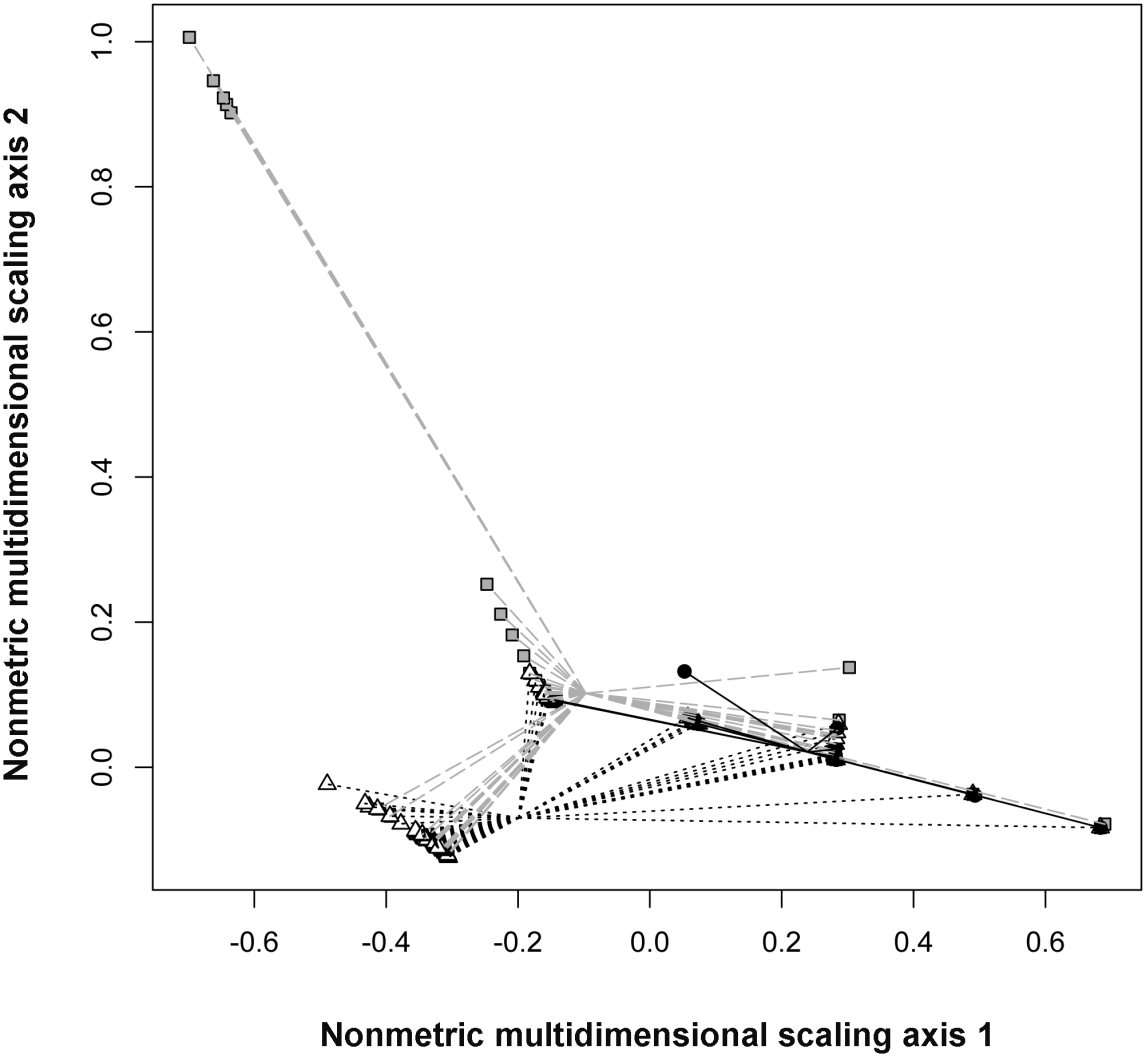
Ordination plot of microhabitat used by the three anurans in NT and WA. The plot shows the first two axes of a Nonmetric Multidimensional Scale analysis for microhabitat use of *Rhinella marina* (black circles), *Litoria caerulea* (triangles) *and L. splendida* (squares). Solid black lines connect all microhabitats used by *R. marina* with the centroid of all observations for the species. Hatched grey lines and hatched black lines connect microhabitats used by *L. caerulea* and by *L. splendida,* with the respective centroids for each species.

Overall, then, the three anuran species differed in microhabitat use (Qb = 69.9, P<0.0001). The habitat use of cane toads differed significantly from that of both frog species (toads vs. *L. caerulea,* Qb = 60.2, P<0.0001; vs. *L. splendida*, Qb = 18.8, P<0.0001; Figure 5). Toads used a narrower range of heights and fewer substrate types than did frogs (Figures 2,3,5). Green Treefrogs and Magnificent Treefrogs did not differ significantly in microhabitat use (Qb = 16.7, P = 0.398; Figures 2,3,5). The ordination plot shows high similarity in microhabitat use by the two treefrogs, but occasional similarity between toads and Green Treefrogs also (Figure 5). Similarity in microhabitat use among species also shifted with time (higher by night than by day: interaction Qb = 9.3, P<0.0001), and was higher in anthropogenically disturbed areas than in natural environments (interaction Qb = 8.19, P = 0.0268), especially at night (interaction type of environment vs. time of the day, Qb = –2.26, P<0.0001).

Microhabitat overlap between toads and the two frog species was generally lower than expected by chance, except in disturbed environments in WA at night (where overlap was high, but not statistically different from that expected by chance: Table 1). At the WA site, pairwise indices showed low overlap between all species during the day, and high overlap between the two frog species at night in disturbed areas. Overlap in microhabitat use among species tended to increase at night, especially in anthropogenic environments (Table 1).

**Table 1:** Pianka Index of microhabitat overlap among invasive cane toads *Rhinella marina* (RM), and native frogs *Litoria caerulea* (LC) and *L. splendida* (LS) in Australia.

Area	Environment	Period	ObservedIndexRM-LC	ObservedIndexRM-LS	ObservedIndexLC-LS	Observed indexall species(if differentfrom pairwise)	AverageSimulatedindex (variance)	P
NT	Anthropogenic	Day	0.18	-	-	-	0.58 (0.047)	0.05
		Night	0.13				0.60 (0.020)	<0.0001
	Natural	Day	0.00	-	-	-	0.55 (0.032)	<0.0001
		Night	0.11	-	-	-	0.59 (0.026)	0.001
WA	Anthropogenic	Day	0.14	0.0	0.56	0.23	0.64 (0.008)	<0.0001
		Night	0.62	0.66	0.96	0.74	0.65 (0.006)	0.099
	Natural	Day	-	0.0	-	-	0.57 (0.019)	<0.0001
		Night	0.42	0.69	0.69	0.60	0.67 (0.005)	<0.0001

### Nearest Neighbor Analyses

Most of the anurans we found were close to other anurans; only 17 at the NT site, and 41 at the WA site, were more than 50 m from another individual of one of our three study species (13%). The identity of species that formed the nearest neighbor pairs depended on the site (χ^2^ = 192.96, df = 5, P<0.0001), type of environment (χ^2^ = 47.78, df = 5, P<0.0001), time of encounter (χ^2^ = 13.27, df = 5, P = 0.021), if the frogs were exhibiting breeding activity (χ^2^ = 96.95, df = 5, P<0.0001), PC1 (χ^2^ = 16.06, df = 5, P = 0.0068), and distance from water (χ^2^ = 34.35, df = 5, P<0.0001, Tables S3 and S4 in File S1). The nearest neighbor was most likely to be a conspecific, except for Green Treefrogs at the WA site (Table 2,). Nearest-neighbor ‘pairs’ of Green Treefrogs were often recorded in natural areas (Table 2), whereas ‘pairs’ of toads were found more often in anthropogenic areas (Table 2). Nearest-neighbor ‘pairs’ of Green Treefrogs were often found late at night (mean time 2120 h) than ‘pairs’ of toads, and of Magnificent Treefrogs (mean time 2025 h). ‘Pairs’ of frogs (including both conspecifics and heterospecific pairs) were often recorded during breeding activity, and often near the water. Frogs encountered serendipitously during the daytime were all in shelters, with a conspecific as their nearest neighbor (*L. caerulea* n = 11, *L. splendida* n = 34).

**Table 2:** Percentage of encounters of nearest neighbours according to species in the two studied areas.

Pair of species/Area	RR	RC	CC	SR	SC	SS
NT	40.4	11.7	47.9	-	-	-
WA	27.6	12.1	10.3	7.5	16.1	26.4
Type of Environment						
Natural	13.6	12.7	58.5	2.5	1.7	11.1
Anthropogenic	43.2	11.5	21.8	3.0	7.7	9.8

R = *Rhinella marina*; C = *Litoria caerulea*, S = *L. splendida*.

#### Solitary vs. aggregated anurans

**Figure 6:**
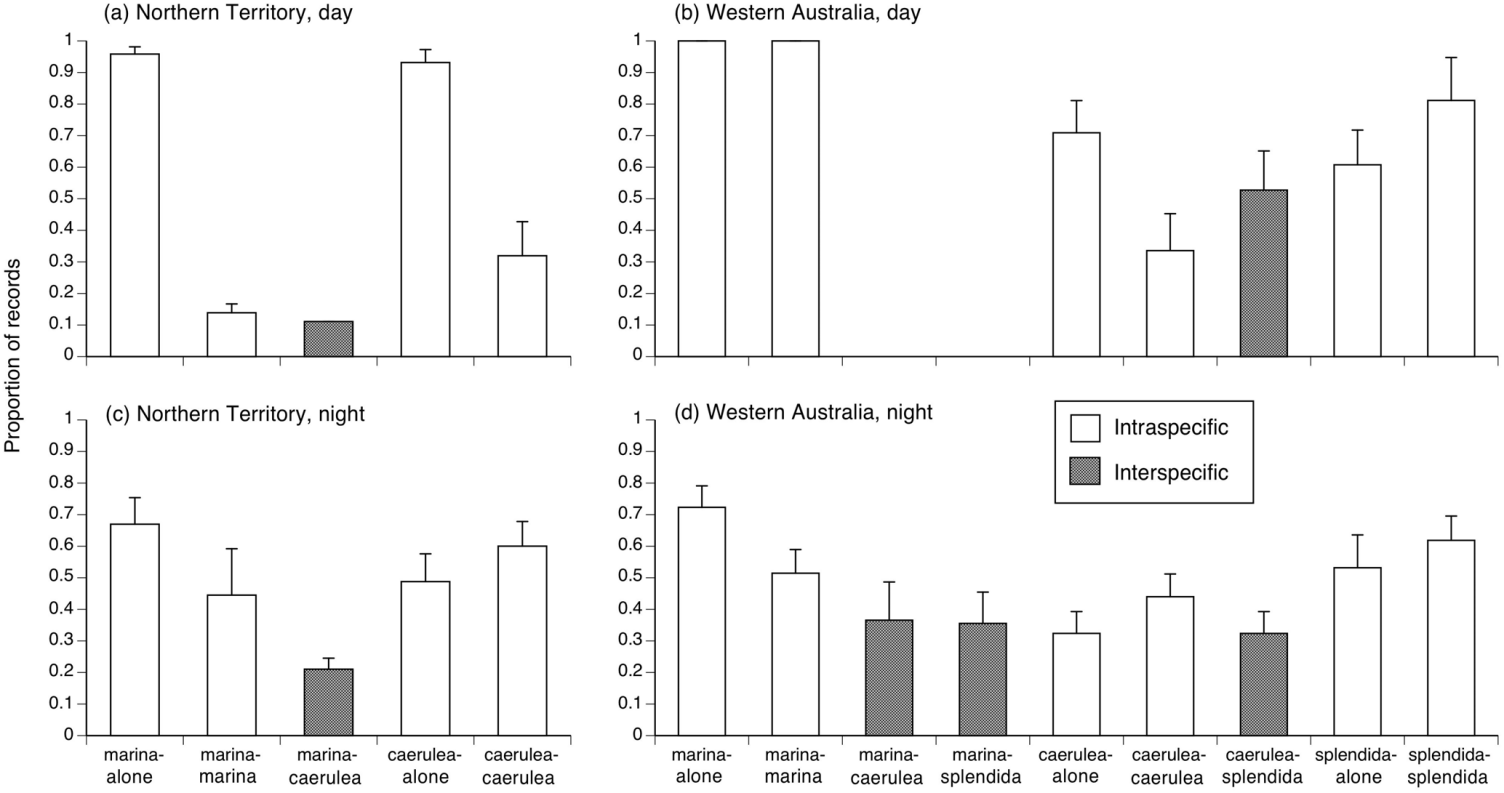
Proportion of encounters of pairs of nearest neighbor species within radio-tracked anurans. caerulea = *Litoria caerulea,* marina* = Rhinella marina,* splendida = *L. splendida*. The panels show data for diurnal habitats in the Northern Territory (a) and Western Australia (b), and for nocturnal habitats in the Northern Territory (c) and Western Australia (d). Graphs show mean values and associated standard errors. Nearest-neighbour pairs that contain two different species are shown in stippled format.

Whether or not a radio-tracked anuran was found within 50 m of another anuran (of our study species) depended on the time of day, and differed between the two regions. By day at the NT site, toads and Green Treefrogs were more likely to be found solitary than to be found close to heterospecifics or conspecifics (χ^2^ = 20.7, df = 4, P = 0.0004; Figure 6). However, that pattern was no longer significant at night (χ^2^ = 9.28, df = 4, P = 0.0544; Figure 6). By day at the WA site, toads were more likely to be found solitary, whereas treefrogs were likely to be found close to other frogs (χ^2^ = 31.9, df = 6, P<0.0001; Figure 6). Similarly, toads were more likely to be found as solitary by night, than were treefrogs (Figure 6).

#### Distance to the nearest neighbor

**Figure 7:**
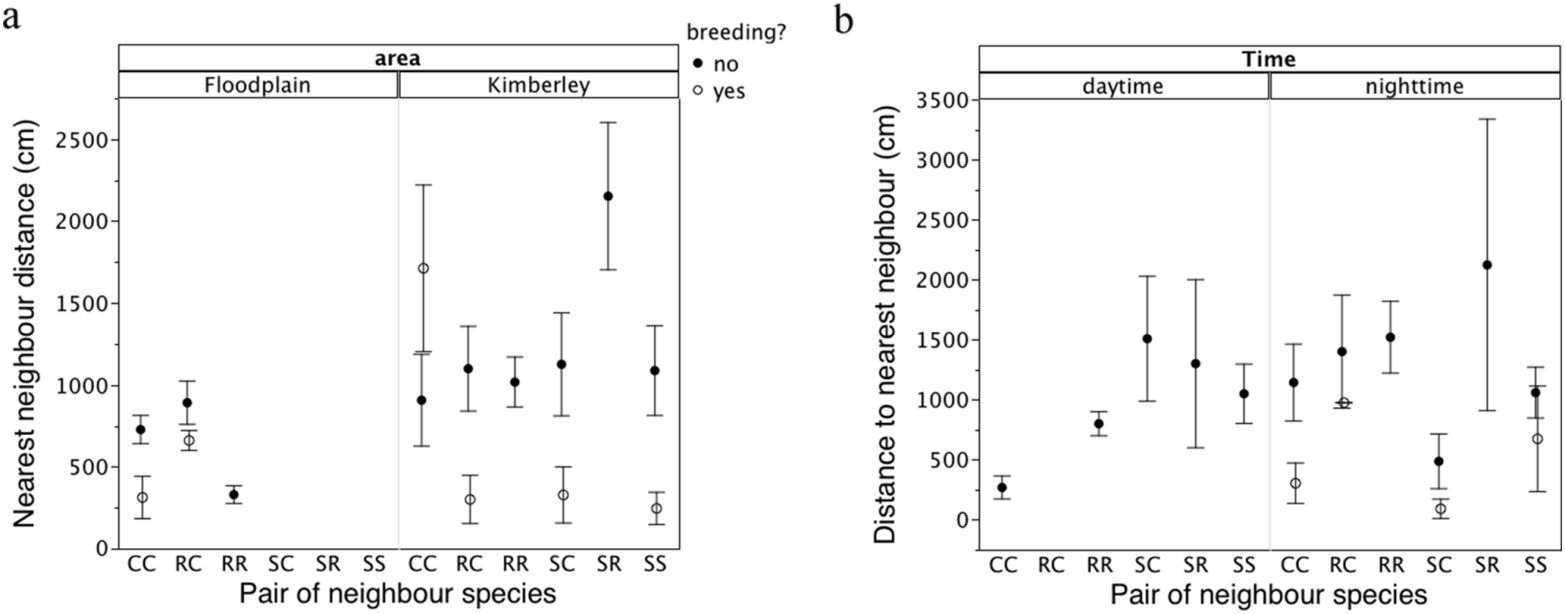
Average distance to the nearest neighbor of anurans. Data show patterns according to the pair of species involved, study site, and occurrence of breeding activity in frogs, at (a) night for surveyed frogs and (b) by day and night for radio-tracked frogs. Bars are standard errors. R = *Rhinella marina,* C = *Litoria caerulea,* S = *Litoria splendida.*

For the surveyed anurans, the distance to the nearest neighbor depended on the pair of species involved (χ^2^ = 57.21, df = 5, P<0.0001), the study site (χ^2^ = 62.53, df = 1, P<0.0001), time of encounter (χ^2^ = 4.65, df = 1, P<0.031), air temperature (χ^2^ = 35.38, df = 1, P<0.0001), rainfall in the previous two days (χ^2^ = 18.51, df = 1, P<0.0001), and whether or not the frogs were breeding (X^2^ = 33.18, df = 1, P<0.0002, Tables S3 and S4 in File S1). Toads were closer to each other at the NT site than at the WA site (Figure 7a). At the WA site, the greatest distances between nearest-neighbors occurred between toads and Magnificent Treefrogs, and toads were closer to breeding Green Treefrogs than was the case at the NT site (Figure 7a). Diurnal data from occasional encounters were too limited to include in the general analyses. At the WA site, we found seven Green Treefrogs together inside a toilet bowl in a house (zero distance), and found groups of n = 6, 12 and 16 Magnificent Treefrogs in caves (average distance to adjacent frog = 75.9±26.3 cm SE, n = 34, Table S3 in File S1).

For radio-tracked anurans, the distance to the nearest neighbor depended on the interaction term between the pair of species involved, and the time of the day (χ^2^ = 9.51, df = 4, P<0.05, Table S5 in File S1). At night, the distance between anurans was strongly affected by breeding activity (χ^2^ = 7.62, df = 1, P<0.006, Table S5 in File S1). The general pattern was similar to that in the surveyed anurans: frogs were close to other frogs (especially conspecifics), whereas toads tended to be solitary. The distances decreased if frogs were breeding (Figure 7b, Table S5 in File S1). The shortest mean distance recorded in the radio-tracked individuals was between Green Treefrogs and Magnificent Treefrogs, especially when breeding. The radio-tracking data also revealed that during the day, frogs were closer to conspecifics than to heterospecifics (Figure 7b, Table S5 in File S1).

## Discussion

The degree of overlap in microhabitat use between a native taxon and an invasive species sets an upper limit to the probable impact of the invader on the native. If the two species live in different places, they are unlikely to eat each other, compete for food with each other, or transfer parasites or diseases to each other. Previous analyses of microhabitat overlap between invasive toads and native frogs have relied upon broad qualitative comparisons [31]. Habitat overlap has been used to predict impacts of predation by invasive species [32] and competition between invaders and native taxa [33], [34]. This approach has not been used to explore the risks of parasite transfer from invaders to native species, but may provide useful insights for conservation managers seeking to minimize potential effects of introduced diseases. Our detailed data show substantial differences in microhabitat use between invasive toads and two species of native frogs, and a trend for individuals of each taxon to be found closer to conspecifics than heterospecifics. Those divergences reduce, but do not eliminate, the possibility that invasive toads will have significant impacts on native frogs.

It is not surprising to find a greater ecological overlap between the two treefrog species than between frogs and toads. The treefrogs are closely related, and share a broad suite of morphological, behavioral and ecological traits. Most obviously, these two *Litoria* species resemble many but not all Australian hylids in exhibiting numerous adaptations to arboreal life such as long limbs, expanded toepads, and deep extensor muscles of the fingers [35]–[38]. Like many arboreal predators, however, both of these anuran species may spend substantial time on and near the ground, foraging for terrestrial prey items (L. Pizzatto, pers. obs., S. Clulow, pers. comm., [39]). That temporal shift towards terrestrial sites, and use of open areas, bring these large green frogs into close contact with invasive cane toads. The overlap between species is higher in anthropogenically disturbed areas than in natural sites, a trend driven by the greater abundance of cane toads in disturbed habitats [40] as well as the reduced availability of arboreal crevices in treeless areas. In our study, overlap with toads tended to be similarly high for *L. caerulea* and *L. splendida* at night, when frogs were active on the ground, especially in disturbed environments. In natural areas, the frequent use of open ground by both *L. splendida* and toads resulted in a high overlap index (as high as for the two frog species). That index is, however, unrealistically high because although both taxa use open surfaces, macrohabitat divergences between toads and Magnificent Treefrogs decrease actual proximity: frogs were mostly restricted to rocks in the high outcrops whereas toads were mainly seen on lower terrain.

We have no data on the diurnal retreats of Green Treefrogs in the natural areas in WA, where this species was uncommon. In this situation, toads and Green Treefrogs may share shelter sites in an area with broken terrain (where toads can access most available crevices), but not in a site with steep cliffs (which provide abundant sites accessible to treefrogs but not toads). Importantly, although our three study species differ significantly in microhabitat use, individuals of all three species (especially toads) make occasional forays into “non-preferred” microhabitat types. Even occasional movements of this type might be enough to infect a few individuals of the other taxon, which might then spread infection to conspecifics, and thus to heterospecific treefrogs.

How will those overlaps translate into the impacts of invasive toads on native frogs? The answer is complex. For example, the toad’s impact might be manifested via shifts in prey availability for frogs, rather than through parasite transfer. Because both toads and large treefrogs have generalized diets [21], [41], the microhabitat overlap will expose them to similar but not identical prey types. Cane toads tend to consume many small insect prey items, whereas native anurans often target larger prey [31], [42]–[44]. Direct predation by cane toads on treefrogs is unlikely to be important, because cannibalism (and by implication, predation on other anurans) is largely restricted to juvenile toads [45]. The cane toad invasion front is dominated by large individuals [46] that feed primarily on insects. For the same reason, predation by treefrogs on juvenile cane toads is unlikely to be common in invasion-front populations. These large frogs also consume anurans [47] and may well consume occasional cane toad metamorphs, as do other large anurophagous native anurans [48]–[50]. Presumably, however, a capacity for taste aversion learning, as demonstrated in other frogs including *Litoria* [48], [50], will lessen the intensity of such impacts at a population level.

How likely are frogs to be infected by the parasites carried by cane toads? To answer that question, we need data on topics outside the scope of the present study. For example, where do toads and frogs defecate? Such sites may well be highly non-random, depending on the times and places where the animals feed and digest their prey, and their body temperatures while in diurnal retreat sites [51]; and if so, a given level of overall overlap in microhabitat use between species may tell us very little about exposure of native anurans to the parasitic larvae expelled with toad feces. To answer this question, we would need a study that identified defecation sites in the wild, perhaps by feeding fluorescent powder to toads to label their feces [52]. Our preliminary trials show that this method can work, but is very time-intensive; many anuran feces that we found were <50 cm from the frog’s diurnal retreat sites (plausibly, within range of a dispersing infective larva). Other critical issues include how long the free-living worms and infective larvae survive in the soil after hatching in the feces, and how far they disperse. We know that the larvae of *Rhabdias pseudosphaerocephala* can persist for at least a week in moist soil [53], that lungworms passed by *Litoria caerulea* (as well as by toads, their native-range host) are capable of infecting new hosts [54], and transmission can be affected by soil and substrate type (e.g., low in sand, very low in water, L. Pizzatto, unpubl. data; see also [52] for North American *Rhabdias*). Quantifying the dispersal and survival of these tiny larvae in the field poses formidable logistical challenges [53], but may not be impossible [52].

Given these uncertainties, we can only make broad conclusions about the possibility that invasive cane toads will spread their lungworms to the potentially vulnerable *Litoria splendida*. Direct transmission (from toad feces to infection of *L. splendida*) is possible, but probably rare. Our radio-tracking study showed that toads can climb and cross rocky landscapes, and were occasionally found in high outcrops. However, they are not found in very steep cliffs (such as gorges, chasms and cave wall crevices), so their feces will rarely be deposited in the arboreal crevices and perches in which the treefrogs spend most of their time. Physical proximity is greater at night, but whereas frogs were sedentary and philopatric, toads dispersed quickly (Pizzatto et al., unpubl. data). The high mobility of toads may reduce the time that the species spend close to each other. Toads and treefrogs also overlap in the types of waterbodies for spawning; amplecting *L. splendida*, eggs and tadpoles were found in puddles and pools in drying creeks (L. Pizzatto, unpubl. data), the same type of waterbodies used by toads [21]. However, unlike toads, treefrogs call away from water and move to the spawning site only when amplexed (L. Pizzatto, unpubl. data). The frogs may not need to return to waterbodies to rehydrate as frequently as do toads [55], and the lungworm larvae does not survive well in water or in saturated soils [56]. The most plausible mechanism for direct transmission of parasites involves treefrog use of open ground at night, in anthropogenetically disturbed areas also frequented by toads.

Transmission of parasite larvae from cane toads to Magnificent Treefrogs may be more likely to occur via the third species (Green Treefrogs) than directly from the toads to *Litoria splendida*. The commensal anuran *Litoria caerulea* overlaps significantly with cane toads in nighttime microhabitat use in disturbed areas (above), and is capable of developing and maintaining a lungworm infection [16], [17]. This species also exhibits some diurnal overlap with toads and shares habitats with its congener *L. splendida*, including diurnal aggregations in arboreal crevices. If the frogs defecate inside or near these crevices, parasite transfer would be facilitated. That is, parasite transfer from toads to Green Treefrogs, and thence to Magnificent Treefrogs, may occur in areas where *L. spendida* occurs in sympatry with both *L. caerulea* and cane toads. The broad distribution of *L. caerulea* [20] means that it is virtually ubiquitous across the wet-dry tropics, but it is relatively uncommon in the pristine rocky habitats preferred by *L. splendida* (L. Pizzatto and C. Both, pers. obs.). Shared use of diurnal refuges by the two treefrog taxa may be increased by anthropogenic disturbance, because of reduction in the availability of other suitable retreat-sites.

Other critical issues that warrant more study involve the effects of *Rhabdias pseudosphaerocephala* infection on *Litoria splendida*. Laboratory trials show rapid mortality of juvenile frogs [17], but we have no data on the fate of adults. If infected adult treefrogs cease moving around and feeding, and rapidly die, the chances of them passing on the parasite to conspecifics may be greatly reduced. On the other hand, adults may survive long enough to allow spread of the parasite [10]. Infection trials in outdoor enclosures could usefully evaluate this possibility.

Predicting parasite transfer from one host type to another by *Rhabdias* species is difficult; some taxa within this genus are highly host-specific whereas others exploit a wide range of hosts [57]. Toads have invaded the region occupied by *Litoria splendida* only within the last three years, and examination of 31 Green Treefrogs collected over that period showed no parasitism by toad lungworms [18]. Dissections of seven *L. caerulea* at two sites where toads have been present for 6 years and >30 years, respectively, also did not reveal any infected frogs [18], and toad lungworms have not caused mortality in experimentally infected adult Green Treefrogs [54]. Broadly, then, our results are encouraging. Invasive cane toads are not commonly found close to, or in the same microhabitats as, the vulnerable native treefrog *Litoria splendida*. Some cross-infection is likely to occur, especially in disturbed habitats where numbers of toads and both frog species are highest, and shelter-site availability is lowest (forcing frogs to aggregate in the remaining shelters). However, many parts of the habitat occupied by *L. spendida* are inaccessible to toads, and are rarely used by *L. caerulea*. We cannot totally discount the threat posed by parasite transfer, but the likelihood of transmission from toads to treefrogs is expected to be low.

## Supporting Information

File S1 **Supporting Tables.** Proportion of records of each anuran species per microhabitat type for each area and environment type (Table S1), microhabitat used by the three anuran species (Table S2), nearest neighbor species and distance at daytime (Table S3) and nighttime (Table S4) for non-tracked individuals and radio-tracked individuals (Table S5) in the Northern Territory and in the Kimberley, Western Australia.
(XLS)
